# Dwell Time Distributions of the Molecular Motor Myosin V

**DOI:** 10.1371/journal.pone.0055366

**Published:** 2013-02-13

**Authors:** Veronika Bierbaum, Reinhard Lipowsky

**Affiliations:** Theory and Bio-Systems, Max Planck Institute of Colloids and Interfaces, Potsdam, Germany; Wake Forest University, United States of America

## Abstract

The dwell times between two successive steps of the two-headed molecular motor myosin V are governed by non-exponential distributions. These distributions have been determined experimentally for various control parameters such as nucleotide concentrations and external load force. First, we use a simplified network representation to determine the dwell time distributions of myosin V, with the associated dynamics described by a Markov process on networks with absorbing boundaries. Our approach provides a direct relation between the motor’s chemical kinetics and its stepping properties. In the absence of an external load, the theoretical distributions quantitatively agree with experimental findings for various nucleotide concentrations. Second, using a more complex branched network, which includes ADP release from the leading head, we are able to elucidate the motor’s gating effect. This effect is caused by an asymmetry in the chemical properties of the leading and the trailing head of the motor molecule. In the case of an external load acting on the motor, the corresponding dwell time distributions reveal details about the motor’s backsteps.

## Introduction

The molecular motor myosin V is a dimeric protein with two identical motor domains or ‘heads’, each of which has a nucleotide binding pocket for the hydrolysis of ATP. The motor transduces the free energy released from ATP hydrolysis into discrete mechanical steps along actin filaments [1], [2]. The properties of these steps have been characterized by changing the ATP concentration and an external load in various single-molecule and chemokinetic experiments [2]–[10] including living cells [11]. The details of the motor’s mechanical steps have been investigated using sophisticated single-molecule techniques [12]–[15]. Moreover, the motor’s motion has directly been visualized through AFM imaging [16].

In a single forward step, the motor unbinds its trailing head from the filament, moves this head forward by 72 nm, and rebinds it to the filament in front of the other head. Since the latter head stays at a fixed filament position, the motor’s center-of-mass is displaced by 36 nm during such a step. This directed motion requires the coordination of the ATP hydrolysis by the two motor heads. It is generally believed that this coordination involves the following motor states and transitions. For most of its time, the motor molecule dwells at a fixed filament position with ADP bound to both heads. The sequence that leads to a forward step starts with ADP release from the motor’s trailing head followed by ATP binding to this head, while the motor’s leading head remains in its ADP state. The different ADP release rates for the trailing and leading head, that we will describe as gating, are thus essential for the coordination of the two heads. However, it has not been possible, so far, to directly measure these two rates for double-headed myosin V. Experiments on single-headed myosin V indicate that resisting and assisting load forces lead to rates that can differ up to 100-fold [8], [9], [17], [18]. In a double-headed molecule, intramolecular strain leads to opposite forces on the two heads of the motor. Therefore, the experiments on single-headed myosin V imply that the different release rates will depend on force as well. In the present study, we will refer to the difference of ADP release rates as gating. Note that the latter term is used with a slightly different meaning by different authors.

This paper is closely related to our previous work, where we have discussed a network description for myosin V that captures the motor’s stepping properties as a function of external control parameters such as nucleotide concentration and external load force [19]. The step velocity of myosin V depends on the concentration of ATP, and decreases with decreasing [ATP]. For a load force that opposes the motor’s stepping direction, the load decreases the motor’s velocity, until this velocity vanishes at the stall force 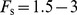 pN [3], [4], [6], [7]. For resisting loads that exceed the stall force, the motor exhibits a ratcheting behaviour, i. e., it steps backwards without being much affected by the ATP. For assisting loads, the step velocity of the motor is independent of the load force. In our previous study [19], the motor’s motion was described by a chemomechanical network that includes both chemical reactions, provided by the binding and release of nucleotides, as well as two mechanical stepping transitions, both of which have the same step size (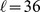 nm). The stepping properties of the motor for both assisting, sub- and superstall forces, were described by three different motor cycles, a chemomechanical, an enzymatic, and a mechanical step cycle. Furthermore, the gating effect was incorporated by differing ADP release rates of the molecule’s leading and its trailing head.

In this paper, we will determine the ratio of the two ADP release rates by analyzing the dwell time distributions as measured for the double-headed motor. We deduce this ratio, termed gating parameter, through comparison of the three cycles discussed in [19] with the experimental dwell time distributions that are available for myosin V [4], [7], [10]. In this way, our work is embedded into the framework of branched chemokinetic networks that have been addressed predominantly in the context of kinesin [20], [21]. Our aim is to directly relate the experimentally determined chemokinetic parameters of myosin V such as the binding and release of nucleotides to the dwell time distributions as measured in single-molecule experiments with double-headed myosin V.

**Figure 1:**
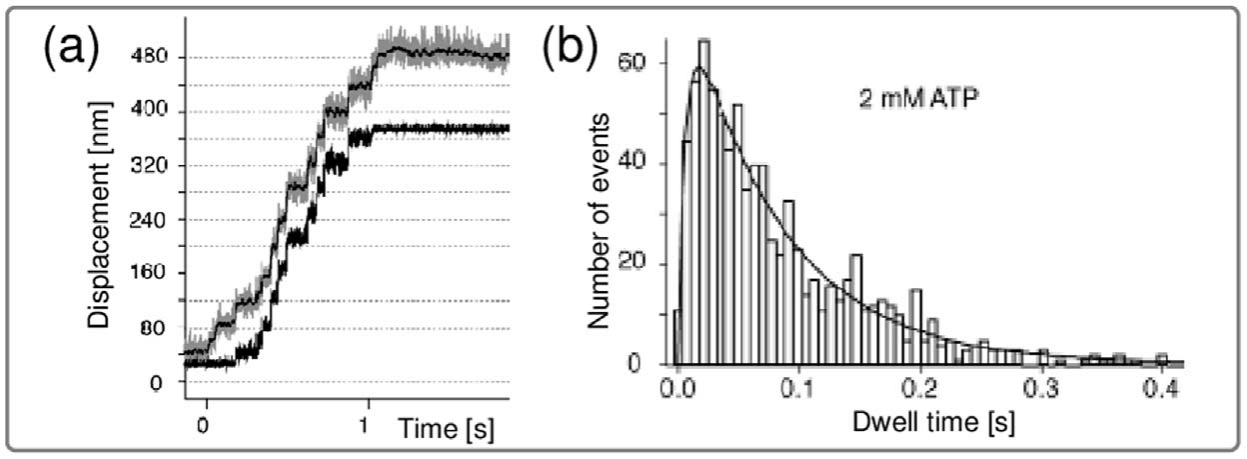
(a) Typical stepping trajectory, i.e., spatial displacement as a function of time and (b) dwell time distribution of myosin V, adapted from [4]. (a) In single-molecule experiments with a feedback loop, the data are monitored under constant external load. Hence, the distance between the bead monitoring the motor’s motion (upper gray trajectory, with the thin black line showing a filtered curve) and the trap center (black trajectory) remains constant. (b) Dwell time distribution of myosin V for saturating [ATP]. The solid line is a fit from Ref. [4] that involves two exponential functions with decay rates 150/s and 12.5/s.

In single-molecule experiments that involve double-headed myosin V, the motor’s steps are monitored through the motion of a bead attached to the stalk of the motor. The evaluation of a stepping trajectory, as shown in Fig. 1, leads to a distribution of its dwell times during which the motor sojourns between two steps. These dwell times provide information about the molecule’s chemomechanical mechanism and have been computed for kinesin [22] and for complex networks of myosin V [23]. For kinesin, it is difficult to measure the dwell time distribution because of the motor’s fast kinetics [24]. For myosin V, however, the slower motion allows to experimentally resolve the overall shape of its dwell time distribution. The network representation for myosin V as introduced in Ref. [19] and used here is based on the experimentally observed separation of time scales between mechanical and chemical transitions [13], [25]. A similar time scale separation has been observed for conventional kinesin [24] and used to construct chemomechanical networks with several motor cycles [21], [26], [27].

This paper is organized as follows. We give a brief overview about network representations for molecular motors and the formalism for the calculation of the dwell time distributions using Markovian dynamics. We first describe the motor’s kinetics by a single chemomechanical cycle that is dominant for external loads 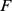 below the stall force 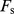, as follows from our previous study [19], in which we used a more complex three-cycle network. We then calculate the motor’s dwell time distributions for various nucleotide concentrations, in very good agreement with experimental data. The use of a network based on few parameters allows to quantify the influence of nucleotide binding and release rates onto the dwell time distributions. Second, taking additional pathways from the more complex network into account, we quantitatively determine the motor’s gating effect. To address force dependent dwell time distributions, we discuss the range of external loads, for which the uni-cycle network applies. Our approach enables us to determine separate distributions for forward and backward steps, and we gain information about the motor’s backward steps through comparison with experimental data. Finally, we summarize and discuss our results.

## Methods

### Network Representations

In general, the stepping properties of molecular motors can be described through network representations with discrete chemomechanical states of the motor supplemented by Markovian dynamics.

**Figure 2:**
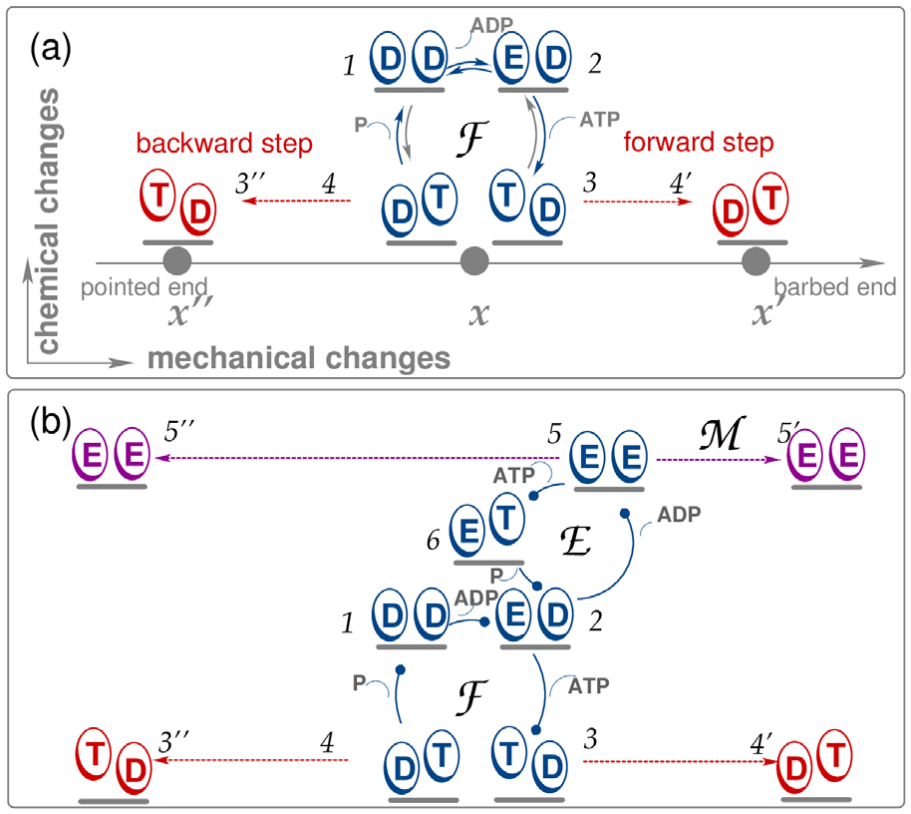
Chemomechanical networks based on the nucleotide states of the two motor heads at site 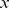, with chemical transitions shown as solid blue lines. The motor can reach states at the neighbouring sites 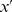 and 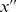 through mechanical transitions (dashed lines). The motor’s step velocity can be calculated by periodically repeating the networks at site 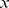 along the spatial coordinate. (a) Uni-cycle network for myosin consisting of the chemomechanical cycle 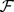. Dashed red lines show mechanical transitions along the filament coordinate 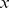, which emerge from the state TD into the forward and from state DT into the backward direction. Solid lines refer to chemical transitions. The arrows indicate the direction of the transition, and infrequent transitions are shown in grey. This uni-cycle network applies to forces below the stall force 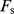. (b) Three-cycle network introduced in [19] that captures the myosin’s stepping properties for both sub- and superstall load forces. The network includes ADP release from the leading head and additional forward and backward mechanical transitions for forced stepping (dashed violet line), with the dots pointing into the direction of hydrolysis. In addition to the network cycle 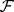, two cycles 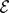 and 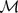 are present. While the enzymatic cycle 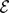 contains only chemical transitions, the mechanical cycle 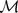 consists only of the mechanical transition 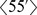. Thus, a spatial displacement can arise by means of the network cycle 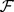 (dashed red lines) or the mechanical cycle 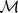 (dashed violet lines).

As explained in previous studies [19], [26], [28], the network for a double-headed motor contains, in general, many chemical states that differ in the occupation of the two heads by nucleotides. It is interesting to note that each of these chemical states represents a branching point of the networks. As shown in Ref. [19], the behavior of myosin V as observed in single-molecule experiments can be described by reduced networks with four chemical states as in Fig. 2(a) or with six chemical states in Fig. 2(b). In these networks, the motor moves along a discrete, one-dimensional coordinate 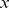 towards the barbed end of the actin filament. The binding sites along the filament are separated by the motor’s step size 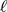. At each site 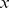, the motor can undergo several chemical transitions that lead either to the hydrolysis or the synthesis of one ATP molecule. These transitions connect the motor’s states that are defined by the chemical composition of its two heads. Each head can contain bound ATP (T) or ADP (D), and it can be empty (E), such that a combination of these states of the motor’s leading and trailing head determine, together with its position, its *chemomechanical* state. To determine the dwell time between two steps of the motor, we consider a network with all chemical states at a given lattice site 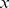 with the states 3′′ and 4′ at neighbouring sites *x*′′ and *x*′. A chemical transition 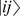 from state 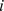 to state 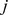 involves the binding or release of ATP, ADP, or P, while the mechanical transitions 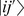 and 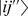 correspond to forward and backward steps of size 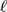.

Throughout this work, we use networks with absorbing boundaries, i. e., network representations that include all motor states at lattice site 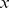 and are truncated at the neigbouring sites 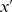 and 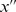. The step velocity of the motor can be obtained when the network cycle 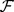 is periodically repeated along the filament, see Fig. 2(a). The network that captures the stepping properties for the experimentally accessible range of load forces as discussed in [19] is shown in Fig. 2(b). It is an extended version of the network in Fig. 2(a), with two additional cycles 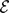 and 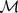. These two cycles become dominant for the motor’s motion in a range of forces that exceed the stall force of the motor. The network contains two stepping transitions, 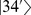 in 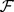 and 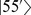 in the cycle 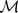. From this network, the motor’s step velocity can be deduced by using multiple copies of the network, see [19]. Here, we focus on the uni-cycle network shown in Fig. 2(a), that, as a sub-network of the three-cycle network, describes the motor’s motion for a restricted range of external loads. This approach allows us to analytically determine the dwell time distributions. Moreover, a direct connection between the chemical binding and release rates of the motor and the dwell time distributions for various nucleotide concentrations can be established. To extract additional information in particular about the gating effect, we return to the more complex network in Fig. 2(b).

The chemomechanical network in Fig. 2(a) describes the stepping behaviour of myosin V in accordance with experimental studies [4], [9] for forces that do not exceed the stall force 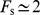 pN of the motor. We will elucidate the dependence on external load in detail further below. The motor starts from the DD state with ADP bound to both heads, releases ADP from its trailing head to attain state ED, binds ATP to its trailing head (TD), and performs a forward step, during which both heads interchange their position (DT). Hydrolysis at the leading head leads to state DD, and, in this way, a trajectory connecting two chemically equivalent states completes the chemomechanical cycle 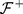 (DD 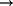 ED 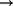 TD 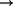 DT 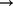 DD). The reverse cycle 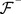 (DD 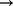 DT 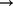 TD 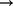 ED 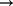 DD) would lead to a backward step DT 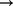 TD coupled to ATP synthesis. The cycles 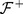 and 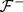 form the network cycle 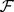.

In vitro experiments are typically performed for relatively low concentrations of ADP and P, which implies that both ATP synthesis and the reverse cycle 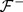 are strongly suppressed. In addition, the rate of ATP dissociation from the trailing head of the myosin V motor, which is part of the reverse cycle 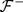, is very small, as discussed in detail in [19]. Backward steps are still possible, however, even for the relatively simple network displayed in Fig. 2(a) since a backward step may occur immediately after a forward step corresponding to the sequence TD 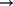 DT 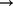 TD. The latter sequence of transitions is very unlikely in the absence of load but becomes more probable with increasing load force [19].

### Gating and Mechanical Details of the Myosin V Step

Before we discuss the actual network dynamics, let us briefly review some molecular details of myosin V, which will be important in order to relate our theoretical results to experimental observations. So far, we have emphasized the uni-cycle network 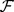. In this network, the release of ADP takes place at the molecule’s trailing head. If the DD state were ‘symmetric’ with respect to ADP release, the probability to release ADP from the leading head would be equal to the one from the trailing head. It is, however, generally agreed that the rates of ADP release are different for the leading and the trailing head [8], [9]. Thus, in order to describe the gating effect in an explicit manner, we need to consider the three-cycle network displayed in Fig. 2(b).

The different ADP release rates from the heads of myosin V are thought to arise from internal strains that the heads experience when they are simultaneously bound to the filament [9]. In this case, one head is subject to a positive internal force and the other to a negative one. Experiments with single-headed myosin V constructs have shown that the ADP release rate depends on the direction of the external load imposed onto the molecule [18]. When both heads are bound to the filament, the motor experiences an internal strain arising from the elastic properties of its lever arms, that corresponds to a force acting on both heads in opposite directions. To what extent this strain is distributed in the double-headed motor, is, however, not a priori clear. The step of myosin V consists of a large, directed swing of its lever, called power stroke, and a diffusional search of the free head for the next target. The elastic energy provided for the power stroke is induced by the hydrolytic reaction taking place at the myosin head. How this elastic energy is distributed in the different chemical states of the motor heads, however, remains unclear. In single-headed molecules, the kinetic properties of the power stroke have been characterized in detail [15], [29]. The stroke is induced through a conformational change, that affects the position of the motor head on the filament, and rotates the lever arm. This conformational change is assumed to affect the ADP-bound state of the motor [15]. In a double-headed molecule, the elastic energy required for the stroke leads to a strained position of myosin V, as deduced from AFM images, which reveal a bending of the molecule’s leading lever [16], thereby changing the internal force acting onto the motor heads. In this way, both the gating and the power stroke have an effect onto the steps of the motor. The substeps of myosin V have been monitored in various experiments [6], [7], [13], [25], with different substep numbers and step sizes. Keeping these observations in mind, we will combine the putative substeps of myosin V into a single step, and discuss the limitations of our approach along with the dependence of the dwell times on an external load.

Another property that is not fully understood is the gating effect. In an experimental study that involves mutants of kinesin-1, the possible causes of the gating effect are elucidated through single-molecule techniques [30]. There, the authors conclude that the gating in kinesin-1 arises through both intramolecular strain and steric effects. Due to the step size of 36 nm of myosin V, which is large compared to the 8 nm step size of kinesin, steric effects for gating are likely to play a minor role for myosin V. For myosin V, both Refs. [5] and [9] conclude that the intramolecular strain leads to an increase in the ADP release rate at the molecule’s rear head and to a decrease of the ADP release rate at the front head. Comparison with the data in [18] that test single heads as a function of force support the conclusion that ADP release from the front head is strongly reduced, while the release at the rear head is only moderately enhanced.

We characterize the gating effect by using an ADP release rate that is measured in chemokinetic experiments [3], and impose the asymmetry through reduction of the ADP release rate at the leading head. Even though we use a specific network here, our approach is applicable to any network description for myosin V that allows for ADP release from both heads of the molecule, such as the one proposed in [28]. However, the parametrization will, in general, differ for different networks, especially with respect to the force dependence of transition rates. Let us now turn to the formalism that allows to compute the dwell time distributions for myosin V.

### Markov Chains

The probability distribution for the time between two successive steps of the motor is governed by a random walk that has one or more absorbing boundaries. This approach corresponds to a first passage problem on a specific network [31], and is closely related to the methods used in [22], [23]. The process starts at a fixed site 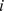 at 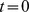 and is stopped when an absorbing state 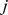 is reached. For a Markov chain that consists of two states, an initial state 0 and an absorbing state 1 connected through the transition rate 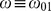, the probability distribution is exponential, which applies to myosin V for superstall resisting forces.

In the trajectories observed in single-molecule experiments, the dwell times between two successive steps correspond to random walks whose dynamics are determined by the underlying chemomechanical network. These random walks start directly after a mechanical step and are terminated after another mechanical step. In addition, these states also terminate the random walk when a step is taken through the mechanical transition. A Markov chain that corresponds to a closed network thus consists of a piece of that network that contains all chemical transitions at a lattice site 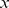 and is terminated at the two neighbouring sites 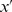 and 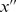, see Fig. 2(a). Thus, the latter two states are *absorbing* states of the network, while the remaining states are *transient*.

For a given Markov chain 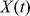 with 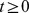 and 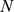 states, let the first 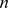 states be transient and the remaining 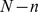 states be absorbing. We denote the conditional probability for the process to dwell in state 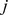 at time 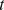 given that it started in state 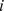 at time 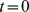 by 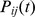. The corresponding master equation reads.
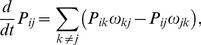
(1)where 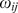 is the transition or jump rate from state 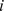 to state 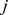. These rates have the general form
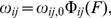
(2)where 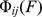 accounts for the force dependence [26]. For better readability, we omit the prime to indicate the spatial coordinate in both the transition rates 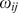 and the functions 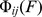. For chemical rates, we have
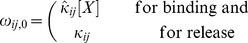
(3)of a nucleotide species X, as appropriate for dilute solutions. For the step rates in 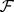, 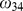 and 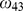, we have
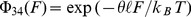
(4)for a forward and
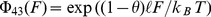
(5)for a backward step with parameter 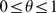 in accordance with the balance conditions from nonequilibrium thermodynamics [27].

In principle, all chemical transition rates may depend on force but this force dependence is difficult to estimate. The force dependence of the binding or release of a specific nucleotide in a complex macromolecule such as a motor head cannot be accounted for by basic approaches such as reaction rate theory. Our minimal approach is thus to neglect the putative force dependence of the chemical rates, unless such a dependence is needed to describe the experimental data. In agreement with experimental studies [9], [18], we thus concluded in [19] that solely the binding rates 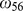 and 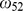 decrease with resisting loads, see section S. 2 of Text S1 for details of the parametrization. A force dependence of these two rates is sufficient to describe the stepping behaviour of myosin V for all three regimes of external load. Thus, we take all chemical rates to be independent of force both for the cycle 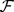 within the three-cycle network in Fig. 2(b) and for the single-cycle network in Fig. 2(a).

The steady state solution to the master equation is given by 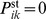 for any transient state, because the walk will eventually always end up in an absorbing state. For an absorbing state 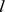, 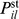 is equal to the probability for being absorbed in 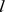 given that the walk started in 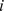, see [22].

The dynamics of the process prior to absorption is identical to the dynamics of an unrestricted Markov process. This means as long as the process does not end up in an absorbing state, its behaviour is identical to that of a closed network: Being in a given state, the process is not influenced by the absorbing states, until the process is terminated. Before reaching an absorbing state, the random walk proceeds with an exponentially distributed waiting time in every transient state 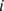,
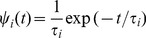
(6)with an average dwell time 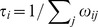. The process starts in a state 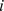, sojourns in each state according to the probability
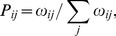
(7)until it is eventually absorbed in state 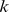. The dwell time of the process is given by the shortest time it takes to arrive in any absorbing state 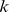 given that the walk started in 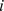, see section S. 1 of the Text S1. To describe trajectories from single-molecule experiments, we are interested in all walks that, as mentioned before, start in a state directly after a mechanical transition. This transition can consist of a forward or a backward step, which implies two possible initial states. In addition, as another mechanical step either in the forward or backward direction terminates the process, we also have two possible absorbing states. In order to distinguish between the subsets of dwell times that arise from forward and backward steps, the conditional probability density distribution 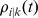 is required. This distribution governs the subset of walks that start in 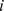 and are absorbed in 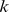, and thus refers to the absorption into a specific state 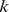. The conditional probability density distribution 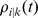 is defined as



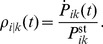
(8)It is given by the time-dependent derivative of the probability, 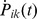, rescaled with the steady state probability for absorption, 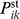. To determine 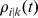 via Eq. (8), we explicitly solve the master equation, to obtain the time-dependent transition probabilities 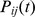, and thus 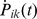. The corresponding steady state solution follows by integration,
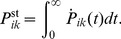
(9)Prior to discussing the explicit form of the dwell time distributions, let us note that in case of a network that does not contain *any* absorbing states, the corresponding master equation can be rewritten in terms of flux differences or excess fluxes 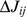 from state 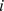 to state 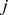,
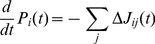
(10)with the excess fluxes 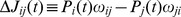 and transition rates 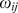. The step velocity of the motor is related to the flux through the mechanical transitions of the network in the steady state with 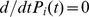. For the network cycle 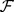 as in Fig. 2(a), the velocity of the motor is then given by
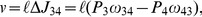
(11)i.e., by the excess flux through the transition 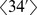.

### Conditional Dwell Time Distributions

Let us calculate the distributions that refer to transitions connecting two subsequent forward or backward steps, 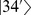 and 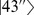, or a backward following a forward step and vice versa, 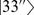 and 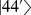. Hence, the four distributions have the initial states 3 and 4, and the absorbing states 3′′ and 4′, respectively. In the chemomechanical cycle 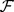, the rates for ATP dissociation and P binding in the case of [P]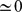 are very small, 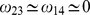, in accordance with the experimental conditions in [4], [7]. For simplification, we set these rates equal to zero in our calculations, such that the pathway 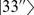 vanishes in the network in Fig. 2(a). For the network in Fig. 2(b) that consists of three cycles, we use the values for the transition rates 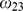 and 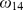 as determined in Ref. [19], while the remaining rates within 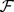 are identical for both networks. The steady state probabilities read.
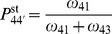
(12)

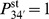
(13)

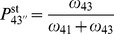
(14)

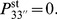
(15)With the use of Eq. 8, the dwell time distributions for these four conditional steps can be explicitly calculated and compared to experimental data. As shown in [22], the distributions that refer to the probabilities of taking a forward and a backward step, 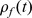 and 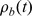, read.



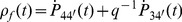
(16)

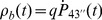
(17)




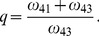
(18)The distribution for all events is given by
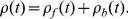
(19)The distributions 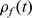 and 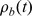 are multi-exponential functions with decay rates 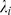, with the tail of the distributions governed by the smallest eigenvalue



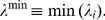
(20)The eigenvalues for the network 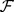 read
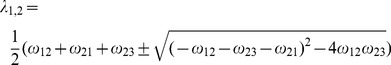
(21)

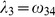
(22)

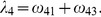
(23)


For small [ADP], the first two eigenvalues reduce to 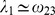 and 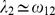.

## Results

### Dependence on Nucleotide Concentrations

In order to compare our results with the experimentally determined distributions reported in [4], [7], we have rescaled the experimental data such that the area covered by the histogram is normalized. Throughout this article, experimental data are shown as green bars, while the total dwell time distributions as obtained from the network shown in Fig. 2(a), appear as solid blue lines. In the experiments in [4], [7], low concentrations of ADP and P were used so that we can put [P] equal to zero as discussed in the previous section. For comparison, we take [P] = 0 in the three-cycle network as well, our results, however, are not altered for [P] = 0.1 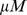, the concentration used in [19]. As ADP binding has more impact on the motor’s motion [2], [32], we use, if not indicated differently, a small concentration of [ADP] = 0.1 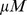 in our calculations for both the single-cycle and the three-cycle network. We have also performed calculations for zero ADP concentration and have checked that the precise value of [ADP] does not alter the distributions in any significant manner.

**Figure 3:**
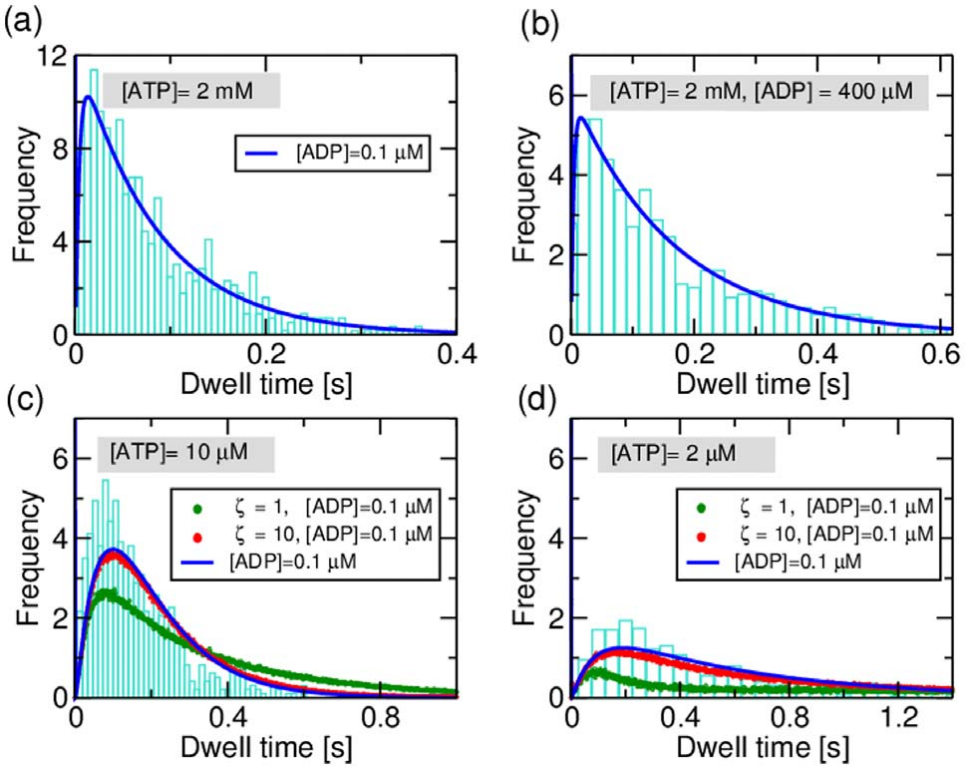
(a–d) Dwell time distributions for different concentrations of ATP and ADP, with [P] = 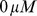 as discussed in the text. Comparison of distributions calculated using the uni-cycle network in Fig. 2(a) (blue solid lines) with experimental data (green bars) from [4]. Insets: Concentrations that apply to both experimental data theoretical curves are shown in the gray panels, while parameters specific to the theoretical results are given in the framed panels. In (a), (c–d), the experimental concentration of ADP is believed to be negligible. For saturating [ATP], (a,b) the dwell time distributions for the uni-cycle network (blue line) agree with those for the network shown in 2(b), for all gating parameters 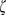 (data not shown). The symbols show simulated data for the network in 2(b) without gating (green circles) and gating with a 10-fold decelerated ADP release from the motor’s leading head (red circles).

Fig. 3 shows the total distribution of dwell times, 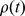, for 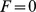 and different nucleotide concentrations using the transition rates shown in Table 1 and the experimental data from [4]. The transition rates for the three-cycle network are given in Ref. [19]. For 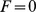, [ATP] = 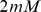, and small [ADP], see Fig. 3(a), our results (blue lines) are in good agreement with the data. We have 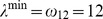 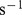, and the tail of the distribution reflects the rate of ADP release. With addition of 400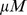 [ADP] (Fig. 3(b)), the distribution broadens significantly, which reflects the inhibiting effect of ADP on the motor’s motion, a fact experimentally well established [2], [6], [32]. For limiting [ATP] (Fig. 3(c, d)), the step velocity is, in the absence of ADP, governed by the rate of ATP binding.

**Table 1:** Transition rates for the uni-cycle network.

binding rates, 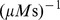		release rates, 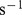		step rates, 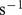	
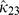 (ATP)	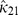 (ADP)	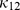 (ADP)	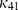 (P)	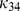	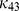
0.9*	4.5**	12*	250*	7000^†^	0.65^‡^

Transition rates for the network displayed in Fig. 2(a) for 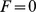, as determined experimentally in [3] (*) and [4] (**), from simulations [35] (^†^), and earlier work [19](^‡^).

The network in Fig. 2(a) contains no transition where ADP is released from the leading head. The gating effect has to be taken into account for networks that involve the transitions DD 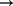 DE or ED 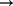 EE. The latter transitions constitute *leaks* from the simple network in Fig. 2(a). In order to address the gating effect, we also considered a more complex network that allows for ADP release from the leading head, as shown in Fig. 2(b). It contains an additional forward and backward stepping transition 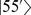 and 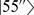 that is active in the regime of superstall resisting forces, see section S. 2 of Text S1. Networks that include ADP release for both the leading and the trailing head, may be supplemented by the simplifying assumption that these rates do not differ. Indeed, the dwell time distributions that are obtained from the network shown in Fig. 2(b) do agree with the experimental data for high concentrations of ATP without any gating.

Let us describe the gating effect by the ratio 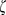 between the ADP release rates from the molecule’s leading and trailing head, i. e.,
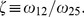
(24)


In the case of limiting [ATP], neglecting the gating effect by assuming equal rates of ADP release for both heads, i. e, 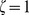, leads to discrepancies between the experimental data and simulated dwell times (green circles in Fig. 3(c, d)) for the network in Fig. 2(b). These discrepancies for low [ATP] can be understood because the ATP binding transition 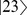, which is rate-limiting for the motor’s kinetics, competes with the transition for ADP release from the leading head, 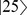. The motion is not affected as long as competing transitions in the network have a small probability compared to ATP binding. In the absence of a gating effect, the rate for ADP release is 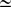 10-fold higher compared to the ATP binding rate at 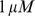 [ATP], which leads to less mechanical steps through the stepping transitions 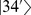 and 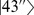. This would result in longer dwell times and hence in a broader distribution than the one observed experimentally. The width of this distribution is primarily determined by the gating parameter 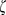 and decreases with decreasing 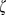.

For the chemomechanical network in Fig. 2(b) which includes the transition ED 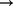 EE, the red circles in Fig. 3(c, d) show the simulated dwell times that are obtained for gating parameter 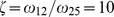, which is in the range of 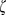 where we find best agreement of the simulated dwell times with the experimental data [5], [9].

**Figure 4:**
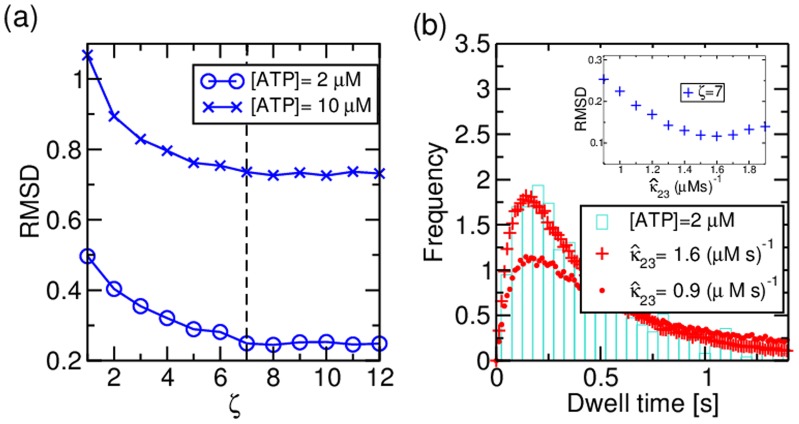
(a) Root mean square deviation RMSD between the experimental data (green bars in Fig. 3 (c, d)) and the simulated dwell time distributions for the three-cycle network as a function of the gating parameter 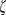 for [ATP] = 10 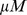 (crosses) and [ATP] = 2 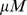 (circles). A lower deviation indicates an improved agreement between the experimental values and the distributions that result from the three-cycle network. For both concentrations, the RMSD decreases with increasing 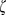, until it saturates for 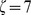, as indicated by the dashed line. The fluctuations for large values of 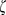 arise from the variance in the simulations. The solid lines serve as a guide to the eye. (b) In case of a variable ATP binding rate 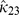, the agreement between the simulated dwell time distributions (symbols) and the experimental data (green bars) is further improved. The agreement is optimal for 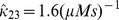 (red crosses), and is significantly improved in contrast to the distribution based on the experimental value of 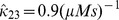 (red circles). The inset shows the RMSD as a function of 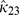, illustrating the minimal deviation for 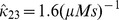



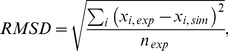
(25)where 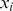 are the dwell times for experiment and simulation, respectively, and 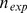 is the number of bins for the experimental data (green bars in Fig. 3(c, d)). Note that we have adjusted the bin size of our simulations to the experimental bin size 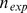 for comparison. For both concentrations, the RMSD decreases with increasing 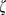 and saturates for values 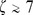. To maintain the forward stepping of myosin V, ATP binding by the trailing head and ADP release from the leading head compete for small concentrations of ATP. For 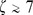, the ATP binding rate is sufficiently large compared to the ADP release from the leading head, and the RMSD saturates. Because all other parameters used for the description of the myosin V velocity are derived from experimental data (in the limit of 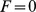), the value of 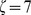 should be regarded as a lower bound for the gating parameter 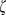.

To determine the optimal gating parameter 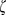, we compared the experimental dwell time distributions and the ones obtained from the three-cycle network for different values of 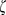. Fig. 4(a) shows the root mean square deviation RMSD between experimental dwell times and simulated ones as a function of the gating parameter 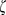 for limiting concentrations of ATP, [ATP]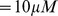 and [ATP]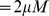. The root mean square deviation has been calculated between the simulated dwell times and the experimental ones as.

The agreement between the calculated dwell time distributions and the experimental data can be further improved by treating the rate 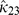 of ATP binding in the chemomechanical cycle 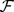 as a fit parameter. Fig. 4(b) shows the dwell time distribution for [ATP]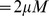 for an ATP binding rate of 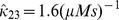, which provides the best fit for fixed gating parameter 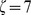. The inset in Fig. 4(b) shows the RMSD as a function of a variable ATP binding rate 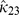, which exhibits a minimum at 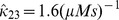. Let us note that with varying 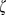, the location of the minimum is shifted marginally within a range that falls below 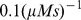. As can be infered from Fig. 4 (b), the agreement between the calculated dwell time distributions and the experimental data can be improved by considering the rate of ATP binding as a fit parameter. For the ATP binding rate 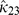, we have used a value of 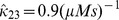 as reported for actin-bound myosin V in chemokinetic experiments with myosin V [3]. The values for ATP binding estimated from single-molecule experiments cover a range of 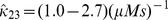 [4], [33]. For 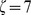, the best fit of our simulations to the data leads to an ATP-binding rate of 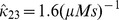, which lies well within this range.

### Dependence on External Load

Before addressing the dwell time distributions of myosin V subject to an external load, let us discuss the motor’s step velocity as a function of external load. The corresponding force-velocity relation is needed to clarify *(i)* the range of external loads where the description via the network formed by the cycle 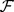 is valid and *(ii)* the set of experimental data that can be evaluated within our theoretical framework.

Using the transition rates as given in Table 1, we calculate the velocity
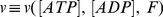
(26)of the motor via Eq. 11. The velocity depends on the external load force 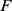 and the concentrations of [ATP] and [ADP] through the transition rates 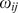.

In the following, we will distinguish three different regimes of external load 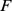: (I) assisting and small resisting forces, where 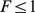 pN; (II) forces close to the stall force with 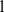 pN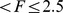 pN; and (III) large resisting forces with 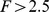 pN.

**Figure 5:**
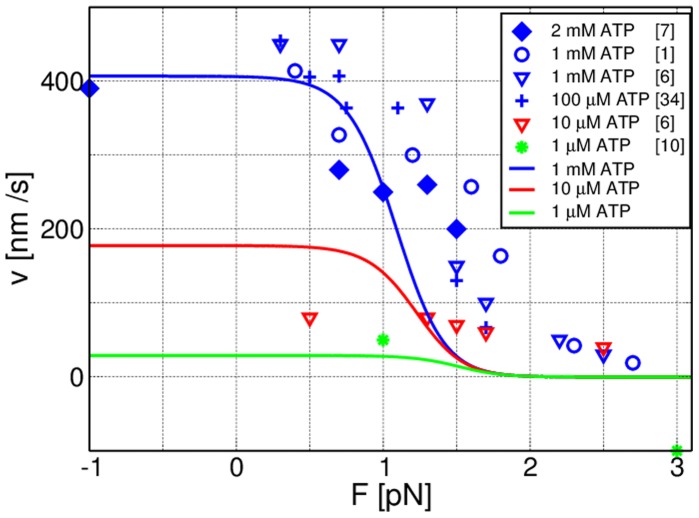
Motor velocity 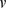 as a function of external load 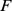 for the network formed by the cycle 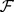 (lines) compared to experimental data (symbols) for varying [ATP]. In the experiments, the concentrations of ADP and P are believed to be rather small. In the calculations, we consider the limit of [ADP] = [P] = 0.

Fig. 5 shows the motor velocity as calculated from the single-cycle network in Fig. 2 (a) with periodic boundary conditions for different concentrations of ATP, with [ADP] = [P]
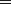
0 and the corresponding experimental data reported by various groups [1], [6], [7], [10], [34]. The experimental values for the stall force cover a range of 1,6–2,5 pN [1], [6], [7], [10], [34], while 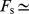 2 pN for the network studied in Ref. [19]. Note that the three-cycle network description of the myosin V motor in Ref. [19] appropriately captures the ratchet mechanism of myosin V observed in [10] for large resisting forces (regime (III)), while the single-cycle description based on the network cycle 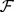 is not valid in this load regime.

For saturating [ATP]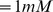, comparison with experimental data shows good agreement for small resisting forces in regime (I). In regime (II) around the stall force 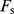, however, there is a discrepancy between the theoretical results and the experimental data. Since our parametrization is solely based on the parameter 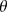 to account for the force dependence of the step rates 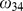 and 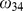, the molecular details of the step are not included in our description. Hence, the motor’s dwell time distributions cannot be compared to experimental data for forces that are close to the stall force. Let us point out that, in addition, the variation in experimental data restricts the possibility to compare all the dwell time distributions that have been measured as a function of external load. The velocities in Ref. [7], shown as solid blue diamonds in Fig. 5, correspond to the experimental dwell time distributions given therein. The distributions are available for forces 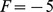 pN, 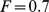 pN, 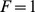 pN, and 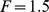 pN from Ref. [7], and for 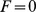 pN from Ref. [4]. Moreover, the dwell time distributions for 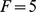 pN can be found in [7] (used here) and [10].

A comparison of the experimental dwell time distribution with the theoretical one is meaningful if the corresponding velocity correctly reproduces the data, which is the case for 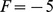 pN and 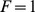 pN. The distribution for 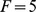 pN will be discussed further below. Since experimental and theoretical results for the velocity do not agree for both 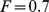 and 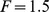 pN, the theoretical and experimental dwell time distributions cannot agree either. The disagreement for 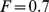 pN reflects the variation in the experimental data and the latter value, 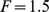 pN, lies in the force regime (II) where the data are not reproduced correctly by our network. Ref. [7] provides a more exact fit to the velocity data for forces that do not exceed 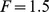 pN by including more force-dependent parameters. It does, however, not lead to the correct prediction of the stall force 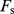. This illustrates the difficulties to correctly describe the complete set of measured dwell time distributions.

**Figure 6:**
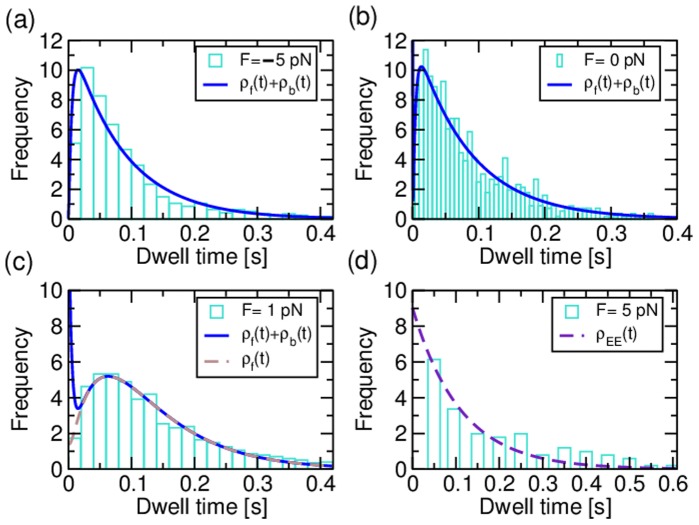
Dwell time distributions (a) for high assisting forces, for 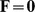 (b) and (c, d) for substall and superstall resisting forces, for 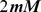 [ATP], 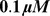 [ADP] and zero [P] with the data from [**4]**, **[7]**. The blue lines show 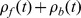 obtained using the single cycle network 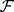 for 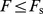. In (c) the distribution of forward steps, 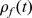 (brown, dashed line) agrees with the data that does not exhibit rapid events as in 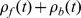. (d) Forced backward stepping for 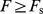 leads to a single exponential decay (dashed violet line) that arises through the mechanical transition 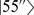 in the network in Fig. 2(b).

The distributions for forces that do not exceed the stall force are shown in Fig. 6(a–c), for 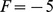 pN, 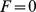 pN and 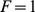 pN. In the presence of an external load, the distributions change through the force-dependent forward and backward stepping rates 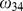 and 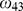 with the force factor 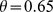. For superstall forces depicted in Fig. 6(d), the motor steps backwards in a forced manner that can be described by the network in Fig. 2(b), which contains the state EE. For 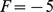 pN, we find good agreement between our theoretical results and the experimental data, as shown in Fig. 6(a). This confirms that for assisting forces, the stepping behaviour is virtually unaltered compared to 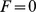, see Fig. 6(b), as observed in [7]. All of the theoretical curves arising from the network in Fig. 2 (a) show a steep decay of rapid events for short times 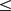 0.01 s. The width of this decay signal increases with increasing resisting load. For a force of 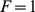 pN as in Fig. 6(c), the width of this peak exceeds the experimental resolution of 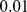 s [7] (blue line). These events of short times are related to the distribution of backward steps, 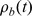, and reflect the increase of the backward stepping rate 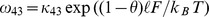 with increasing load. The experimental data in Fig. 6(c) however, agree with the distribution of forward steps, 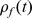 (dashed brown line). The number of backward stepping events observed in [7] might have been insufficient to determine the distributions of 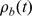. These fast events have also been observed in simulations for single-headed myosin V constructs [23]. Thus, experimental studies would be desirable that address these short dwell times to gain more insight into the mechanical properties of the motor, such as the reversal of its power stroke [15].

In load regime (II) that is close to the stall force, the paramerization with a single force-dependent parameter 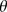 is not sufficient to explain the motor’s dwell time distributions. We have rescaled the theoretical distribution for 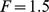 pN in such a way that its maximum agrees with the experimental data in Ref. [7]. The rescaled distribution and the experimental one disagree, which might be due to additional transitions 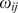, e.g., those that capture sub-steps induced by the motor’s power stroke, as discussed in the context of the gating effect. Since the step velocity of the motor decreases more slowly in the experiments than in our theory, one might speculate that the molecule’s mechanical properties stabilize its chemical activity in the presence of an external force. In part, this stabilization effect has been accounted for by a force threshold in the parametrization of the chemical rates, see section S. 2 of Text S1 and [19], which provides a correct reproduction of the ratcheting behaviour of myosin V but does not affect the mechanical step of the motor in itself. Because of the shortcomings of our model for forces around the stall force corresponding to force regime (II), it seems plausible to expect that effects in the mechanical step rates arising from the motor’s power stroke, have also to be taken into account to describe the slow decrease in velocity with increasing load force.

For high resisting forces, 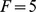 pN, the motor steps in a forced manner, with a single stepping rate as determined in Ref. [19] based on the data in [10]. For superstall forces, the mechanical cycle 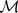 governs the molecule’s motion in force regime (III), such that the motor steps solely through the transition 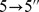. In this case, the dwell time distribution reduces to an exponential function 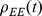 with rate 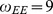/s for 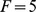 pN, as shown in Fig. 6(d) in agreement with the data.

## Discussion

In this paper, we have focused on a network description of myosin V that consists of only four chemomechanical states, and calculated the dwell time distributions for this molecular motor. Our approach provides a direct relation between nucleotide binding and release rates, that are accessible via chemokinetic experiments, and the dwell times distributions as observed in single-molecule measurements of myosin V. The dwell time distributions obtained from our network description agree with the experimental data for a wide range of nucleotide concentrations, Fig. 3, and substall load forces, Fig. 6(a–c). In case of small ADP concentrations, the tails of the distributions are governed, for saturating [ATP], by the ADP release rate, and by the ATP binding rate for small concentrations of ATP. Comparison with a more complex network (Fig.2(b)) allows us to elucidate the gating effect, see Fig. 3(c, d). In networks that include ADP release from the leading head, ATP binding competes with ADP release from this head. A significant impact on the motor’s step velocity arises once the two transition rates have comparable strength; the motor velocity is reduced by ADP release. The gating effect leads to a regulation of this inhibition through a suppressed rate of ADP release from the motor’s leading head with respect to its trailing head. Through comparison with the experimental data, we quantify the gating effect through an ADP release rate that differs 10-fold for the motor’s leading and the trailing head. In the case of an external load force acting on the motor, we have determined the range of forces that can be described through the network that consists of four states by analysis of the force velocity relation of myosin V, Fig. 5. In addition, we distinguish between dwell time distributions that arise from backward and from forward steps of the motor. For intermediate resisting forces, the experimental data agree with the distribution of dwell times that is associated with forward steps. A peak for short events can be related to backward steps, see Fig. 6(c). Our analysis strongly reinforces the hypothesis that the motor’s motion is governed, for forces up to the stall force, by a simple chemomechanical cycle rather than a complex branched network, which is coordinated by the force-dependent release of ADP. A further step is to relate the information about fast events to the motor’s power stroke.

## Supporting Information

Figure S1
**Repeated version of the network shown in** **Fig. 2(b)** **in the main text, with three network cycles 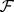, 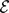 and 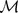.** The stepping transitions in the cycle 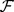 are dominant for forces below the stall force, while steps through the mechanical cycle 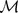 occur for superstall resisting forces, as discussed in [19].(TIF)

Figure S2
**Occupation probabilities 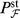 and 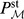 of the network cycles 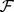 and 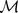 for [ATP] = 2 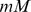 and [ADP] = [P] = 0.1 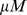.** The chemomechanical cycle 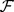 dominates for forces below 1.8 pN and the mechanical cycle 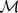 for forces above 2.2 pN. In a transition regime of 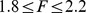 pN, indicated by the horizontal lines, both cycles influence the system.(TIF)

Figure S3
**Dwell time distributions for forces that cover the intermediate regime 1.8 pN 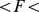 2.2 pN, in a range of 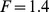 pN to 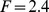 pN, simulated using the complete network from** **Fig. 2(b)** **in the main text.** The nucleotide conditions have been fixed to [ATP] = 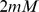, [ADP] = [P] = 0.1 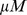. The shape of the distribution resembles, for 1.4 and 1.6 pN, the shape of the distributions for forces that are below these values. The distribution broadens as approaching a vanishing step velocity of the motor at the stall force 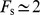 pN, where the sharp peak of short events vanishes and turns into a single exponential distribution, whose slope rises with increasing the load force, as seen for 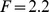 and 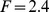 pN. Note that the simulation is based on 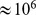 events.(TIF)

Text S1 **S. 1. Absorbing boundary formalism. S. 2. Three-cycle network.**
(DOC)
