# Supplementary figures and images for: Dwell Time Distributions of the Molecular Motor Myosin V
Source: PLoS One. 2013 Feb 13;8(2):e55366. doi: 10.1371/journal.pone.0055366 (PMC3572133; doi:10.1371/journal.pone.0055366)

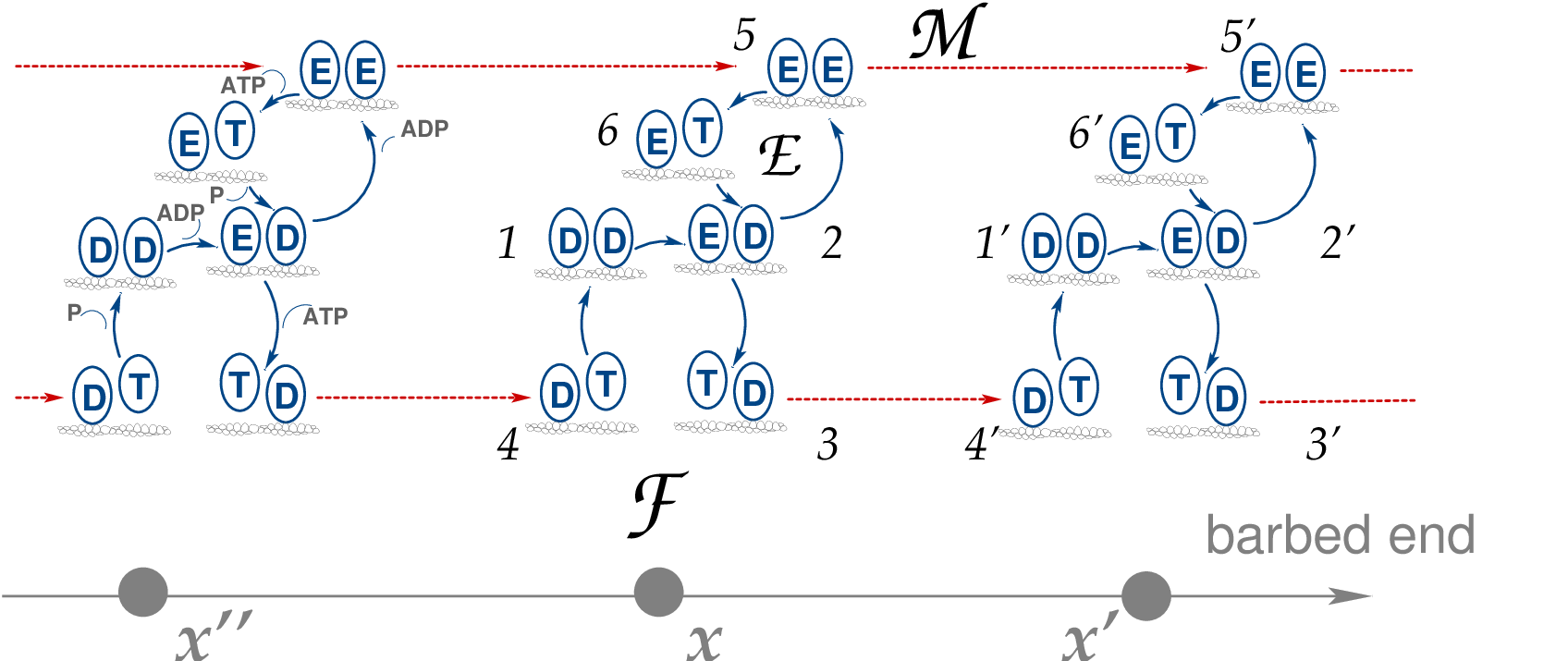

Supplement: Figure S1 — Repeated version of the network shown in Fig. 2(b) in the main text, with three network cycles , and . The stepping transitions in the cycle are dominant for forces below the stall force, while steps through the mechanical cycle occur for superstall resisting forces, as discussed in [19]. (TIF) [file pone.0055366.s001.tif]

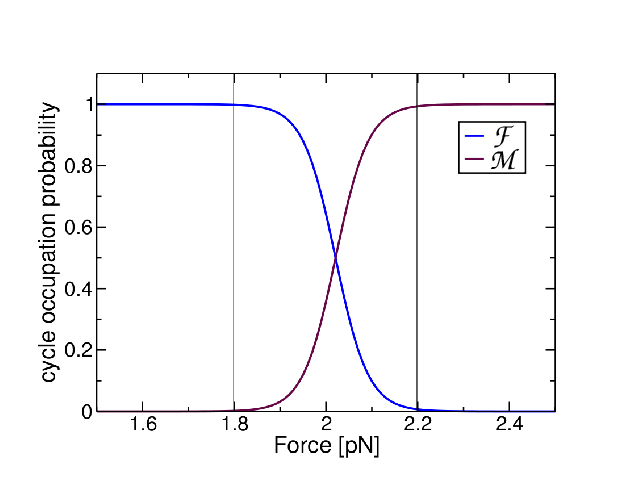

Supplement: Figure S2 — Occupation probabilities and of the network cycles and for [ATP] = 2 and [ADP] = [P] = 0.1 . The chemomechanical cycle dominates for forces below 1.8 pN and the mechanical cycle for forces above 2.2 pN. In a transition regime of pN, indicated by the horizontal lines, both cycles influence the system. (TIF) [file pone.0055366.s002.tif]

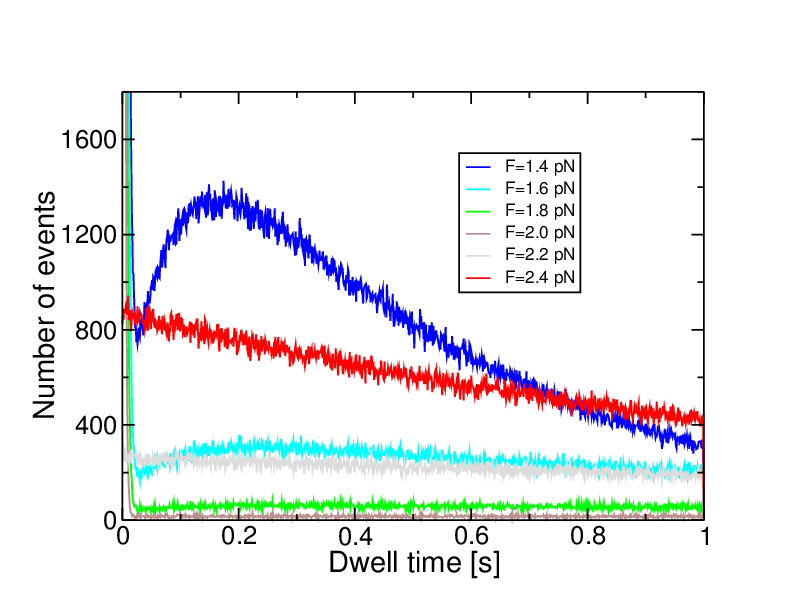

Supplement: Figure S3 — Dwell time distributions for forces that cover the intermediate regime 1.8 pN 2.2 pN, in a range of pN to pN, simulated using the complete network from Fig. 2(b) in the main text. The nucleotide conditions have been fixed to [ATP] = , [ADP] = [P] = 0.1 . The shape of the distribution resembles, for 1.4 and 1.6 pN, the shape of the distributions for forces that are below these values. The distribution broadens as approaching a vanishing step velocity of the motor at the stall force pN, where the sharp peak of short events vanishes and turns into a single exponential distribution, whose slope rises with increasing the load force, as seen for and pN. Note that the simulation is based on events. (TIF) [file pone.0055366.s003.tif]
