# Supplementary material for: Dwell Time Distributions of the Molecular Motor Myosin V
Source: PLoS One. 2013 Feb 13;8(2):e55366. doi: 10.1371/journal.pone.0055366 (PMC3572133; doi:10.1371/journal.pone.0055366)
Supplement: Text S1 — S. 1. Absorbing boundary formalism. S. 2. Three-cycle network. (DOC) [file pone.0055366.s004.doc]

**Supporting Information - Dwell time distributions of the molecular motor myosin V**

Veronika Bierbaum, Reinhard Lipowsky

**1 Theory and Bio-Systems, Max Planck Institute of Colloids and Interfaces, Potsdam, Germany**

**E-mail: bierbaum@mpikg.mpg.de**

**S.1. Absorbing boundary formalism**

In this section, we outline the absorbing boundary formalism for the calculation of dwell time distributions. The formalism of Markov chains and random walks can be found in textbooks such as [1]. We consider a network with $N$ states, with $1...k-1$ transient and $k...N$ absorbing states. The master equation with absorbing boundaries,

$\frac{d}{dt}P_{ij}=\sum_{k\neq j} \left(P_{ik}\omega_{kj}-P_{ij}\omega_{jk}\right)$ (1)

has a steady state solution that is given by $P_{ij}^{\text{st}}=0$, where ‘st’ explicitly refers to the steady state, for any transient state $1\leq i\leq n$, with the normalization condition $\sum_{k=n}^{N} P_{ik}^{\text{st}}=1$.

For an absorbing state $k$, the steady state solution is equal to the probability for being absorbed in state $k$ given that the walk started in state $i$,

$P_{ik}^{\text{st}}=\Pr\{X^{\text{abs}}=k|X(0)=i\},$ (2)

where ‘$\text{abs}$’ stands for absorbing. The dynamics of the process prior to absorption is identical to the dynamics of an unrestricted Markov process. Before reaching a boundary, the random walk proceeds with an exponentially distributed waiting time in every transient state $i$, $\psi_{i}(t)=\frac{1}{\tau_{i}}\exp(-t/\tau_{i})$ with an average dwell time $\tau_{i}=1/\sum_{j} \omega_{ij}$. The process starts in a state $i$, sojourns in each state according to the probability $P_{ij}=\frac{\omega_{ij}}{\sum_{j} \omega_{ij}},$ until it is eventually absorbed in state $k$. The time for absorption in any of the states $n<k\leq N$, when starting in state $i$ is given by the lower bound for the time to reach the respective state,

$t_{i}^{\text{abs}}=\min\{t\geq0,X(t)=k\geq n|X(0)=i\}.$ (3)

This time is referred to as the dwell time of the process.

To obtain the distribution of dwell times, consider the probability that absorption in any of the states $n...N$ takes place prior to a given time $t$, i. e.,

(4)

where the second equality has been deduced in [2, 3]. The corresponding probability density for absorption with an initial transient state $i$ is then given by .

The formalism introduced above refers to the case of absorption in any of the absorbing states $n<k\leq N$. For absorption in a *specific* state $k$, one has to find the subset of those walks that start in $i$ and are absorbed in $k$,

$P_{ij|k}=\Pr\{X(t)=j|X(0)=i,X^{\text{abs}}=k\}.$ (5)

It is given by the fraction of walks that start in $i$, sojourn in $j$ and are absorbed in $k$,

$P_{ij,k}(t)=\Pr\{X(t)=j, X^{\text{abs}}=k|X(0)=i\}$ (6)

$=P_{ij,k}(t)P_{jk}^{\text{st}},$ (7)

with respect to all walks that start in $i$ and are absorbed in $k$:

$P_{ij|k}\equiv\frac{P_{ij,k}(t)}{P_{ik}^{\text{st}}}=\frac{P_{ij}(t) P_{jk}^{\text{st}}}{P_{ik}^{\text{st}}}.$ (8)

Consequently, the conditional probability density distribution $\rho_{i|k}(t)$ that refers to $P_{ij|k}$ is defined as

$\Pr\{t_{i|k}^{\text{abs}}\leq t\}\equiv P_{ik|k}(t)\equiv\int_{0}^{t} \rho_{i|k}(t')dt'.$ (9)

Using Eq. (8) and $P_{kk}^{\text{st}}=1$, one gets

(10)

The conditional and the total probability densities are connected via

$\rho_{i}(t)=\sum_{k=n}^{N} P_{ik}^{\text{st}}\rho_{i|k}(t).$ (11)

To determine $\rho_{i|k}(t)$ with Eq. 10, we explicitly solve the master equation to obtain the time-dependent transition probabilities $P_{ij}(t)$, and thus . The matrix of the transition probabilities that contains the solutions of the unrestricted master equation is given by $P(t)=Q^{-1}\exp\left(-\Lambda t\right)Q.$ Here, $Q$ and $Q^{-1}$ are the matrices in the transformation diagonalizing the transfer matrix **T** of the process, $\Lambda=Q^{-1}TQ$. It is defined as $T_{ij}=-\omega_{ij}$ for $i\neq j$ and $i$, $j$ connected, $T_{ij}=\sum_{i} \omega_{ij}$ for $i=j$, and $T_{ij}=0$ otherwise. $\Lambda$ is a diagonal matrix of the $n$ eigenvalues $\lambda_{i}$ that belong to $T$, and $Q$ is constructed from the eigenvectors of $T$. The derivative of the time-dependent probability distribution to arrive in an absorbing state $k$, , is, in general, given by the probability to jump into that state from a neighbouring site, times the transition rate into the absorbing state. Hence,

(12)

where is an $n$-by-$n$ identity matrix, for an initial state $i$ and an absorbing state $k$.

**S.2. Three-cycle network**

In this section, we compare the properties of the network that consists of three cycles , , and , with the network that is formed by the cycle (Figs. 2(b) and 2(a) in the main text) influenced by an external load force $F$.

Fig. S.1 shows a repeated version of the network with three cycles, that characterizes the motor properties like the step velocity $v$ and the step ratio $q$ for both substall and superstall forces, as shown in earlier work [4]. Here, we focus on different regimes of external load that allow for a description using single cycles, and refer to [4] for details of the network in Fig. S.1 such as the transition rates $\omega_{ij}$. The stepping rates in the three-cycle network that belong to the cycle are identical, with the exception that the exact values for phosphate binding and ATP dissociation were used rather than the limit of these rates being 0. As in Ref. [4], we use the parametrization

(13)

for the force dependence of the chemical transition rates, where $\chi_{ij}$ is a dimensionless parameter and $F'$ is a force scale. The threshold value $F'$ is comparable to the internal strain estimated for myosin V [5]. As shown in Ref. [4], the available single motor data can be described by the three-cycle network in Fig. S.1 ( which corresponds to Fig. 2(b) of the main text) if only the chemical rates $\omega_{56}$ and $\omega_{52}$ are taken to be force-dependent. Thus, we will ignore a possible force-dependence of the chemical rates both for the cycle within the three-cycle network in Fig. S.1 and for the single-cycle network in Fig. 2(a) of the main text.

In the network shown in Fig. S.1, forward steps can arise through the transitions $|34'\rangle$ and $|55'\rangle$, while backward steps arise through the transitions $|4'3\rangle$ and $|5'5\rangle$. As a consequence, the motor's velocity is now given by the fluxes through the transitions $|34'\rangle$ and $|55'\rangle$,

(14)

that arise through the network cycles and .

These cycles are dominant in different regimes of external load. To show this, we consider the cumulative occupation probabilities in the network cycles and as a function of external load force $F$. They are given by and for fixed spatial coordinate $x$. As long as the probability for being in either of the cycles or is 1, mechanical steps arise predominantly through that cycle, while mechanical steps in the other cycle are rare events. Fig. S.2 shows and for both assisting and resisting external forces.

For forces below the stall force $F_{\text{s}}$, the cycle dominates the system, while stepping for high forces is governed by the mechanical cycle . In an intermediate regime of $1.8<F<2.2$ pN, both cycles contribute to the step velocity. The corresponding dwell time distributions of the network arise from the pathways that are possible for any of the combination for the initial states $3,4$, and 5 and the final states $3',4'',5'$ and $5''$. All of these pathways have been taken into account in the simulations for the full network, such that the dwell time distributions $\rho_{f}(t)+\rho_{b}(t)$ contain all possible stepping events.

Fig. S.3 shows the dwell time distributions of all stepping events, $\rho_{f}(t)+\rho_{b}(t)$, for forces in the range of $1.4\leq F\leq2.2$ pN. The distribution that is multi-exponential for $F<F_{\text{s}}$ broadens with increasing load (F=1.4 pN - F=2.0 pN), and flattens out completely as approaching the stall force $F_{\text{s}}$. Thus, the stall force where the motor velocity vanishes corresponds to vanishing motor steps in contrast to frequent sequences of forward followed by backward steps. For forces that exceed the stall force, the distribution rises as a nearly single exponential function (grey and red line), indicating that the mechanical cycle now dominates stepping. This intermediate regime of forces illustrates a ‘switching’ between the two cycles and . For forces below $F\leq1.8$ pN and above $F\geq2.2$ pN, the dwell time distributions arise almost exclusively through the pathways that connect states within the cycles and . For forces $F>2.2$ pN, the network cycle dominates the system. For these forces, the forward stepping rate in this cycle, $\omega_{55'}$, is 0, and stepping arises through the forced backward stepping rate $\omega_{5'5}$. The dwell time distributions can be described by the single network cycle for $F<1.8$ pN. The velocities that correspond to the three-cycle network and the one formed by are virtually identical in this regime of load for saturating concentrations of ATP; they differ by less than 0.1 %.

Let us conclude with further remarks on the gating effect. Without an a priori assumption about the gating effect, one would assume that the ADP release rate for the front and the rear head of the molecule are identical, and hence, $\zeta=1$. As shown in Fig. 3(c, d) in the main text, the motor velocity at low ATP concentrations is suppressed compared to the experimental data. We have checked that other networks, such as the one proposed in [6], do qualitatively exhibit the same behaviour with respect to the gating effect: whenever a transition that contains ADP release from the front head of the molecule is contained in the network, the experimental step velocity can be reproduced for a gating with $\zeta\geq10$, and there is a clear disagreement between data and the theoretical prediction without any gating. The stability of the simple chemomechanical cycle that consists of four states is thus maintained by the effect of gating.

**Figure Legends**

**Figure S. 1.** Repeated version of the network shown in Fig. 2(b) in the main text, with three network cycles , and . The stepping transitions in the cycle are dominant for forces below the stall force, while steps through the mechanical cycle occur for superstall resisting forces, as discussed in [4].

**Figure S. 2.** Occupation probabilities and of the network cycles and for [ATP]=2 $mM$ and [ADP]=[P]=0.1 $\mu M$. The chemomechanical cycle dominates for forces below $1.8$ pN and the mechanical cycle for forces above $2.2$ pN. In a transition regime of $1.8\leq F\leq2.2$ pN, indicated by the horizontal lines, both cycles influence the system.

**Figure S. 3.** Dwell time distributions for forces that cover the intermediate regime $1.8$ pN $<F<$ 2.2 pN, in a range of $F=1.4$ pN to $F=2.4$ pN, simulated using the complete network from Fig. 2(b) in the main text. The nucleotide conditions have been fixed to [ATP]=$2 mM$, [ADP]=[P]=0.1 $\mu M$. The shape of the distribution resembles, for 1.4 and 1.6 pN, the shape of the distributions for forces that are below these values. The distribution broadens as approaching a vanishing step velocity of the motor at the stall force $F_{\text{s}}\simeq2$ pN, where the sharp peak of short events vanishes and turns into a single exponential distribution, whose slope rises with increasing the load force, as seen for $F=2.2$ and $F=2.4$ pN. Note that the simulation is based on $\approx10^{6}$ events.

**References**

1. van Kampen NG (1992) Stochastic processes in physics and chemistry. Elsevier, revised and enlarged edition.

2. Valleriani A, Liepelt S, Lipowsky R (2008) Dwell time distributions for kinesin's mechanical steps. EPL 82: 28011.

3. Liao JC, Spudich JA, Parker D, Delp SL (2007) Extending the absorbing boundary method to fit dwell-time distributions of molecular motors with complex kinetic pathways. Proceedings of the National Academy of Sciences of the United States of America 104: 3171.

4. Bierbaum V, Lipowsky R (2011) Chemomechanical network cycles of the molecular motor myosin V. Biophysical Journal 100: 1747.

5. Veigel C, Schmitz S, Wang F, Sellers JR (2005) Load-dependent kinetics of myosin-V can explain its high processivity. Nature cell biology 7: 861.

6. Astumian RD (2010) Thermodynamics and kinetics of molecular motors. Biophysical Journal 98: 2401.
